# Stable, metastable and unstable cellulose solutions

**DOI:** 10.1098/rsos.170487

**Published:** 2017-08-30

**Authors:** Marta Gubitosi, Pegah Nosrati, Mona Koder Hamid, Stefan Kuczera, Manja A. Behrens, Eric G. Johansson, Ulf Olsson

**Affiliations:** Physical Chemistry, Lund University, Box 124, 221 00 Lund, Sweden

**Keywords:** cellulose dissolution, cellulose aggregation, strong alkali solvents, tetrabutylammonium hydroxide, small-angle X-ray scattering, cellulose regeneration

## Abstract

We have characterized the dissolution state of microcrystalline cellulose (MCC) in aqueous tetrabutylammonium hydroxide, TBAH(aq), at different concentrations of TBAH, by means of turbidity and small-angle X-ray scattering. The solubility of cellulose increases with increasing TBAH concentration, which is consistent with solubilization driven by neutralization. When comparing the two polymorphs, the solubility of cellulose I is higher than that of cellulose II. This has the consequence that the dissolution of MCC (cellulose I) may create a supersaturated solution with respect to cellulose II. As for the dissolution state of cellulose, we identify three different regimes. (i) In the stable regime, corresponding to concentrations below the solubility of cellulose II, cellulose is molecularly dissolved and the solutions are thermodynamically stable. (ii) In the metastable regime, corresponding to lower supersaturations with respect to cellulose II, a minor aggregation of cellulose occurs and the solutions are kinetically stable. (iii) In the unstable regime, corresponding to larger supersaturations, there is macroscopic precipitation of cellulose II from solution. Finally, we also discuss strong alkali solvents in general and compare TBAH(aq) with the classical NaOH(aq) solvent.

## 1. Introduction

Cellulose is the main structuring component in the cell wall of plants and the most abundant biopolymer in the world [1]. It is a linear polymer formed by the repetition of d-glucose building blocks, and the crystal structure of the native cellulose (cellulose I) has been described by a monoclinic unit cell containing two cellulose chains in a parallel orientation [2]. Cellulose may occur also in other polymorphs [3], of which cellulose II is the most thermodynamically stable, and can be obtained via regeneration from solution. Associated with numerous applications, e.g. in papermaking and biofuel industries and even as a textile fibre, the usage of this vastly available polysaccharide instead of traditional resources may lead to lower dependencies on fossil fuels and water supplies and thus a lower impact on the environment. However, to be able to successfully make use of the many benefits of the cellulose polymer, the latter must first be dissolved in a suitable solvent, a problem that has been fraught with many difficulties due to tedious dissolution mechanisms and environmentally hazardous solvents.

Despite its polar structure, cellulose is well known to have an extremely low solubility in water, due to the presence of an extended network of hydrogen bonds and, as recent studies underlined, hydrophobic interactions [4–7]. Such solubility can be enhanced by decreasing or increasing the pH [8,9]. For instance, it has latterly been established that basic solvents containing hydroxide ions may increase the dissolution strength thanks to the strong proton acceptor properties of the hydroxide anion [10], but the exact mechanism of the dissolution is as yet not clearly known. A common solvent for cellulose dissolution has up to now been sodium hydroxide; however, the conditions for dissolution must be stringently controlled [11]. Recent studies have highlighted ionic liquids (i.e. salts that are liquid at room temperature) for the dissolution process [12], and among them tetrabutylammonium hydroxide (TBAH) [13–16], a highly basic, low-viscous ionic liquid, commercially available solvated in different weight percentages of water, TBAH(aq). At temperatures above 27°C, this mixture adopts a purely liquid phase, but at lower temperatures the water molecules form clathrate cages, leading to crystallization [17,18]. The peculiarities yet strengths of TBAH as a solvent [13] make it a good candidate for the dissolution of cellulose in, for example, the fibre wet spinning process. Moreover, as water is commonly used as a coagulation bath in the said process, the study of the phase boundaries of the cellulose/TBAH/water system becomes crucial for the understanding of the fibre formation process.

The aim of this work is to investigate the stability and metastability of solutions containing cellulose in TBAH(aq) using turbidity and small-angle X-ray scattering (SAXS), which give information on the solution stability and the structure at nanometre length scale, respectively. The samples studied in this work range between 0.01 and 0.10 g cm^−3^ microcrystalline cellulose (MCC) in 15–55 wt% aqueous solutions of TBAH. Furthermore, wide-angle X-ray scattering (WAXS) was used to characterize the regenerated cellulose, including fibres obtained by extrusion using a step-motor-driven syringe pump.

This work was thought to extend the study on the stability of MCC in TBAH(aq). In our previous research [14,15], MCC was dissolved in a wide concentration range (0.001–0.10 g cm^−3^) in 40 wt% TBAH(aq), and two different concentration regimes were highlighted, where molecularly dissolved cellulose chains and aggregates were detected at low and high concentrations, respectively. In the present paper, we have extended our studies to a higher TBAH concentration, as well as characterizing solution structure and stability when diluting with water, identifying stable, metastable and unstable solution regimes.

## 2. Material and methods

### 2.1. Materials

MCC (C. Löfgren 2015, personal communication) was purchased from Sigma (Avicel PH-101, DP = 260, particle size 50 µm) and TBAH(aq), originally 40 and 55 wt% in water, was obtained from Sigma-Aldrich.

### 2.2. Sample preparation

Different concentrations of MCC in 40 and 55 wt% TBAH(aq) were prepared by weighing the proper amount of TBAH(aq), transferring it to a glass vial, and adding the cellulose as a last step to avoid sedimentation and clumping at the bottom of the vial. The solvent bottle was prewarmed at 40°C for approximately 1 min prior to weighing to prevent clathrate formation. After the addition of cellulose, the vial was immediately put on vigorous stirring, yielding solutions of various viscosities depending on cellulose concentration. The dissolution time also varied depending on cellulose concentration, on average from approximately 3–5 min (0.01–0.07 g cm^−3^) to 1–2 h (0.07–0.10 g cm^−3^). Lower concentrations of TBAH were prepared by diluting the 40 wt% TBAH aqueous solution with Milli-Q water before the addition of cellulose. The samples containing 0.01–0.06 g cm^−3^ regenerated cellulose were prepared by the dilution of a 0.06 g cm^−3^ regenerated cellulose solution with TBAH(aq).

### 2.3. Regeneration of cellulose

Cellulose was regenerated from a solution of 0.02 g cm^−3^ MCC in 40 wt% TBAH(aq). One hundred ml of solution was prepared as described above in the Sample preparation section, and washed with Milli-Q water and consequently centrifuged for 15 min at 4000 rpm and 20°C for 5 times. The system was subsequently freeze-dried to give 1.62 g of cellulose powder, with a yield of 82%. The obtained cellulose was identified as cellulose II via WAXS (see Results and discussion section).

### 2.4. Turbidity

Data were collected using a Cary WinUV spectrophotometer, at 30 ± 1°C with *λ* = 632.8 nm. Measurements of the transmittance were zeroed for the solvent and taken every 15 s for 60 min. Plastic cuvettes with a path length of 1 cm were used.

Samples containing 0.02 g cm^−3^ cellulose in TBAH(aq) concentrations ranging from 15 to 40 wt% were prepared by adding Milli-Q water to readily prepared solutions of MCC in 40 wt% TBAH(aq) until the required concentrations were obtained and the measurements started within 1 min and lasted for 60 min. Samples of regenerated cellulose (cellulose II) in the concentration range 0.01–0.06 g cm^−3^ in 40 wt% TBAH(aq) were measured within 24 h after preparation.

Cellulose showed no absorption at said wavelength, hence the measured transmittance *T* was considered due only to scattering, and gives the turbidity τ as

2.1 $$\tau =-\frac{\ln T}{l},$$

where *l* is the path length.

### 2.5. Small- and wide-angle X-ray scattering

SAXS and WAXS measurements were performed using a laboratory-based GANESHA instrument (SAXSLAB A/S). The instrument was arranged with a two-pinhole set-up using scatterless slits and the X-rays were generated in a micro-focus source with *λ *= 1.54 Å. The scattered light was detected on a two-dimensional Pilatus detector. All liquid samples were measured at 30°C in quartz capillaries to avoid the formation of TBAH(aq) clathrates, and all experiments were performed *in vacuo*. The SAXS data were collected using two different configurations of the instrument, allowing the scattering spectrum to be collected over a broad *q*-range, 0.003–0.6 Å^−1^. The WAXS experiments were performed using a third configuration, with a *q*-range over 0.3–3 Å^−1^, on MCC and regenerated cellulose powder and on the regenerated cellulose filaments. The scattering vector *q* is defined as

2.2 $$q=\frac{4\pi }{\lambda }\sin\left(\frac{\theta }{2}\right),$$

with *θ* being the scattering angle. For dilute solutions, the SAXS intensity can be written as

2.3 $$I(q)=\frac{c\Delta \rho^{2}M_{w}}{\rho_{c}^{2}N_{A}}P(q)S(q).$$

Here, *c* denotes the cellulose concentration (g cm^−3^), Δ*ρ* = 4.0 × 10^10^ cm^−2^ is the scattering length density difference between cellulose and the solvent, *M*_w_ is the weight average cellulose molecular weight, *ρ*_c_ the cellulose density and *N*_A_ the Avogadro number. *P*(*q*) is the form factor and *S*(*q*) the structure factor.

### 2.6. Fibre extrusion

**Figure 1.**
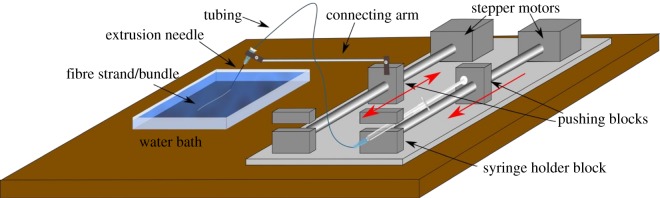
Schematic of the experimental set-up for the fibre extrusion experiments. The motion of the pushing blocks during the experiments is indicated by the red arrows.

The fibre extrusion set-up consisted of a custom-built step-motor-driven syringe pump system with two independently controlled motors (figure 1). The pump set-up was originally designed for microfluidic experiments by Silva *et al.* [19]. One pump was used in the conventional way, driving the solution out of a syringe (500 µl, Hamilton gastight) into a needle (Gauge 21, 0.5 mm in diameter) via a flexible tube (Tygon formulation S-54-HL, 1 mm diameter) with a controllable flow rate *Q*. The needle was fixed to a metal arm in a way that the needle tip was submerged in a water bath. The other end of the metal arm was attached to the pushing block of the other pump. This allowed for a controlled motion of the needle tip within the translational motion range of the pump pushing block (50 mm). For the extrusion of cellulose fibre, the needle tip was repetitively moved back and forth, while the cellulose solution was pumped through the needle tip. This produced a cellulose solution bundle of connected strands in the water bath that precipitated after a few minutes, forming a (wet) fibre bundle. This fibre bundle was left in the water bath for approximately 30 min to wash out the solvent. Afterwards, it was taken out of the water bath and dried at room conditions for several hours. The dried fibre bundle was then stored in an Eppendorf vial before performing scattering experiments. In the no stretching case, the velocity of the moving pushing block *v*_pb_ was equal to the average fluid velocity in the needle, *v*_fluid_ = *Q*/*A*, where *A* is the cross-sectional area of the needle. For stretching with a factor *f*, *v*_pb_*= f v*_fluid_. The flow rate *Q* in all experiments reported here was 10 µl min^−1^.

## 3. Results and discussion

### 3.1. Cellulose in 40 wt% TBAH(aq)

We have, in two recent publications [14,15], characterized not only the dissolution state of MCC, but also dissolving pulp in 40 wt% TBAH. The main conclusions from these papers can be summarized as follows. (i) MCC is molecularly soluble below approximately 0.04 g cm^−3^, as seen by SAXS. (ii) For higher concentrations, up to 0.10 g cm^−3^, SAXS data showed increasing amounts of aggregation. Assuming that cellulose I (MCC) has a higher solubility than cellulose II, it was proposed that the dissolution of MCC may create a supersaturated state with respect to cellulose II resulting in aggregation. Interestingly, the aggregates in this concentration regime appear to be kinetically stable. No coarsening was observed within days. The onset of aggregation at approximately 0.04 g cm^−3^ was ascribed to the solubility of cellulose II.

### 3.2. Cellulose in 55 wt% TBAH(aq)

**Figure 2.**
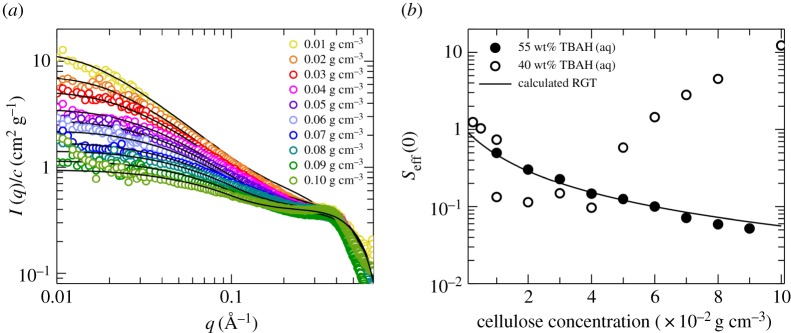
(*a*) SAXS patterns for 0.01–0.10 g cm^−3^ MCC solutions in 55 wt% TBAH(aq). The data are on absolute scale, normalized by cellulose concentration. Solid black lines are model calculations (see text). (*b*) *S*_eff_(0) versus cellulose concentration for 40 and 55 wt% TBAH(aq) solutions. *S*_eff_(0) values from the 55 wt% TBAH(aq) system (filled circles) are obtained from the SAXS data analysis (see text). Data from the 40 wt% TBAH(aq) system (open circles) are taken from [9]. The solid line represents a RGT model prediction (see text).

SAXS experiments were performed on 0.01–0.10 g cm^−3^ MCC in 55 wt% TBAH(aq). The absolute scaled SAXS patterns, normalized by the cellulose concentration, are shown in figure 2*a*.

In the whole range of concentration, the curves exhibit a characteristic feature centred at 0.35 Å^−1^, as already reported for 40 wt% TBAH(aq) [14]. As the concentration of cellulose is increased, a progressive downturn at low *q*-value is observed, with the normalized intensity decreased by approximately one order of magnitude for the analysed concentration range. From such downturn, it is possible to infer the presence of effectively repulsive interactions in the system, which were observed for the 40 wt% TBAH(aq) system only below a cellulose concentration of approximately 0.04 g cm^−3^ [14]. Only at 0.10 g cm^−3^, a slight upturn of the intensity at low *q*-values is notable, indicating the onset of aggregation. We identify this concentration as the solubility of cellulose II. When the concentration of TBAH is increased from 40 to 55 wt%, the solubility of cellulose is increased.

As for the 40 wt% TBAH(aq) solutions [14,15], the scattering patterns were modelled using the form factor of a semi-flexible chain with excluded volume interactions, combined with that of a stiff rod within the persistence length. Such model was implemented considering a core--shell cross section of the latter to describe the feature at 0.35 Å^−1^, which was interpreted as due to a first solvation shell around the cellulose chain enriched in TBAH compared to the bulk solvent.

To describe the repulsive interactions among the polymer chains in solution, a structure factor *S*(*q*) that considers random phase approximation [20,21] was taken into account:

3.1 $$S(q)=(1+\nu P{\left(q)\right)}^{-1},$$

with *ν* being the interaction parameter.

The model parameters used to calculate the scattering intensity were contour length *L* = 1.81 × 10^3^ Å, persistence length *λ*_p_ = 20 Å and *ν* ranging from 1.6 to 20 for the different cellulose concentrations. The calculated $S_{\mathrm{eff}}(0)={\left(1+\nu \right)}^{-1}$ values are reported as a function of cellulose concentration in figure 2*b*.

In the thermodynamic limit at *q* = 0, the structure factor is proportional to the osmotic compressibility and is a measure of the effective interchain interactions. For semi-diluted polymer solutions, the dependence of *S*_eff_(0) as a function of polymer concentration has been calculated by Ohta *et al*. using renormalization group theory (RGT) [22,23]:

3.2 $$S_{\mathrm{eff}}{\left(0\right)}^{-1}=1+\frac{1}{8}\left(9X-2+\frac{2\ln(1+X)}{X}\right)\exp\left(\frac{1}{4}\left[\frac{1}{X}+\left(1-\frac{1}{X^{2}}\right)\text{ln( }1+X\text{) }\right]\right),$$

where *X*, for polydisperse polymers, is given by

3.3 $$X=\frac{B_{2}cM_{w}}{9/16-(\ln(M_{w}/M_{n})/8)},$$

where *c* is the cellulose concentration, *M*_w_ and *M*_n_ are weight and number averaged cellulose molecular weights, respectively, and *B*_2_ is the second virial coefficient. For excluded volume interactions [24], the latter can be written as

3.4 $$B_{2}=\frac{4\pi^{3/2}N_{A}R_{g}^{3}}{M_{w}^{2}}\Psi ,$$

where *R*_g_ is the radius of gyration and *Ψ* is the degree of chain interpenetration, which depends on interactions and can hence be estimated. *Ψ* equals 0 for *θ*-conditions, while it approaches the value 0.24 for good solvents [25]. From the modelling of the *S*_eff_(0) trend as a function of concentration using the RGT, we estimated a *Ψ* of approximately 0.17 and *B_2_* of 1.2 × 10^−3^ cm^3^ g^−2^, with *B*_2_ > 0 being an indication of repulsive cellulose–cellulose interactions in solution. Figure 2*b* compares the so obtained values with the ones calculated elsewhere [15] for 40 wt% TBAH(aq). The overall trend of the previously studied system showed a change in the type of interactions in solution, highlighting the presence of two regimes (repulsive interactions below a concentration of 0.02 g cm^−3^, attractive interactions above 0.04 g cm^−3^). For the higher concentration of TBAH, the whole studied cellulose concentration range lies in the same regime, and a neat improvement of the solvent quality is observed when going from the low cellulose concentrations in 40 wt% (estimated as *Ψ* = 0.04 and *B*_2_ = 6.7 × 10^−4^ cm^3^ g^−2^) [8] to 55 wt% TBAH(aq).

### 3.3. The solubility of cellulose in TBAH(aq)

Assessing the solubility of cellulose in a solvent is non-trivial. First, we need to distinguish between the different cellulose crystalline polymorphs, mainly cellulose I and cellulose II [14,15]. Then there is the general problem that the cellulose source is typically highly polydisperse. Nevertheless, having a number for the cellulose solubility is useful, but it is necessary to take a pragmatic approach and accept that the numbers quoted are essentially estimates.

**Figure 3.**
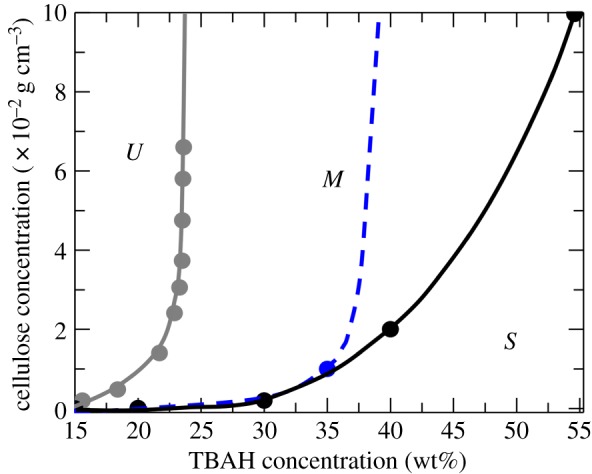
Stability map of the cellulose/TBAH/water system displaying regimes where solutions are stable (*S*), metastable (*M*) or unstable (*U*). The blue dashed line is the estimated effective solubility of cellulose I (MCC). The solid black curve represents the estimated solubility curve of cellulose II.

To determine the solubility of MCC (cellulose I), MCC was gradually added to a TBAH(aq) solution until no more MCC was able to dissolve and the solution remained turbid with undissolved material. The results are presented as an effective solubility curve in figure 3. The solubility increases with increasing TBAH concentration. For less than or equal to 20 wt% TBAH, the MCC solubility is practically zero. At 30 wt% TBAH, it is approximately 0.002 g cm^−3^ and increases to 0.01 g cm^−3^ in 35 wt% TBAH. Then in 40 and 55 wt% TBAH, the solubility appears to be larger than 0.10 g cm^−3^.

**Figure 4.**
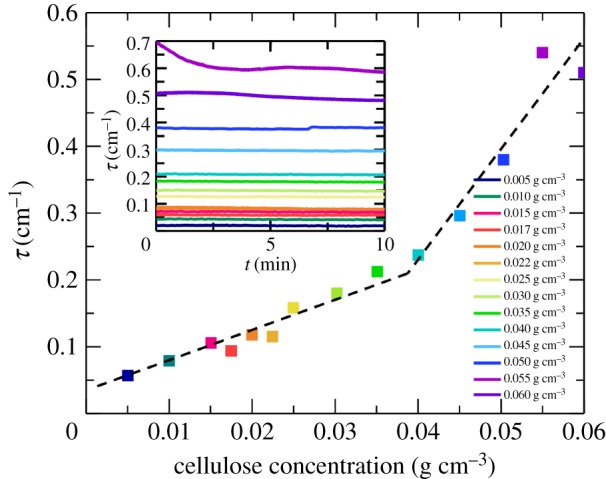
Turbidity values averaged over 10 min for 0.005–0.05 g cm^−3^ regenerated cellulose (cellulose II) solution in 40 wt% TBAH(aq). The dashed line is a guide for the eye.

In the same way, we also determined the solubility of cellulose II. Such solubility was estimated to be 0.02 g cm^−3^ in 40 wt% TBAH(aq), and 0.10 g cm^−3^ in 55 wt% TBAH(aq). To further prove the findings obtained by SAXS, the solubility of cellulose II in 40 wt% TBAH(aq) was inferred by using light microscopy (see electronic supplementary material, figure S1) and turbidity measurements (figure 4).

### 3.4. Coagulation in water

Cellulose can be coagulated from TBAH(aq) solutions by diluting with water. Samples of different cellulose concentrations in 40 wt% TBAH(aq) were gradually diluted with water until the sample turned highly turbid and a macroscopic precipitation of cellulose was observed. The sample compositions marking the onset of macroscopic precipitation are presented in figure 3. Typically, macroscopic precipitation occurs when the TBAH concentration decreases below approximately 23 wt%. We refer to the region where macroscopic precipitation occurs as the unstable region (*U*).

**Figure 5.**
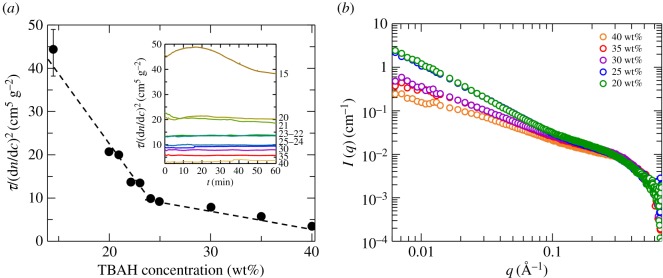
(*a*) Turbidity values averaged over 60 min normalized for the increment of the refractive index for solutions of 2 wt% MCC in 15–40 wt% TBAH(aq). The dashed line is a guide for the eye. Inset: turbidity time trends over 60 min for the same samples. (*b*) SAXS data on absolute scale obtained for 0.020 g cm^−3^ MCC solutions in 20–40 wt% TBAH(aq).

The stability of cellulose solutions at different TBAH concentrations (15–40 wt%) was also investigated by turbidity experiments at a constant cellulose concentration of 0.02 g cm^−3^. Samples of different concentrations of cellulose in 40 wt% TBAH(aq) were diluted with water until a cellulose concentration of 0.02 g cm^−3^ was reached. The turbidity values of the diluted samples were recorded for a period of 1 h. In figure 5*a,* we have plotted the average turbidity, recorded during the 1 h time period as a function of the TBAH concentration. The change in contrast, due to changes in the solvent composition, is corrected for by normalizing the turbidity values with the square of the refractive index increment, (d*n*/d*c*)^2^. The individual turbidity traces are shown in the inset of figure 5*a*.

From 40 down to 25 wt% TBAH, there is a significant, but relatively minor increase of the normalized turbidity, indicating gradual aggregation of cellulose. The aggregation was confirmed by SAXS. In figure 5*b,* we present SAXS data of samples varying the TBAH concentration between 20 and 40 wt%. The increase in the scattering intensity at lower *q*-values is a clear demonstration of cellulose aggregation. Decreasing the TBAH concentration below 24 wt%, a steeper increase in turbidity is observed, and is consistent with an increased aggregation and a transition to unstable solutions.

At 40 wt% TBAH, the 0.02 g cm^−3^ cellulose is below the solubility of cellulose II and the solution is stable. Lowering the TBAH concentration, we reduce the cellulose II solubility below 0.02 g cm^−3^, and this results in aggregation. However, macroscopic precipitation does not occur. As seen in the inset of figure 5*a*, the turbidity has after dilution quickly reached a stable value that does not vary during the 1 h experiment. We identify the aggregated samples in this region as metastable (*M*) or kinetically stable. At 15 wt% TBAH, however, we observe a variation of turbidity in time. First, the turbidity increases indicating continued aggregation or coarsening. Then, at longer times, the turbidity decreases, possibly due to the sedimentation in the sample. We discuss in more detail below the significance of stable, metastable and unstable solutions.

### 3.5. Regeneration of cellulose and fibre extrusion

**Figure 6.**
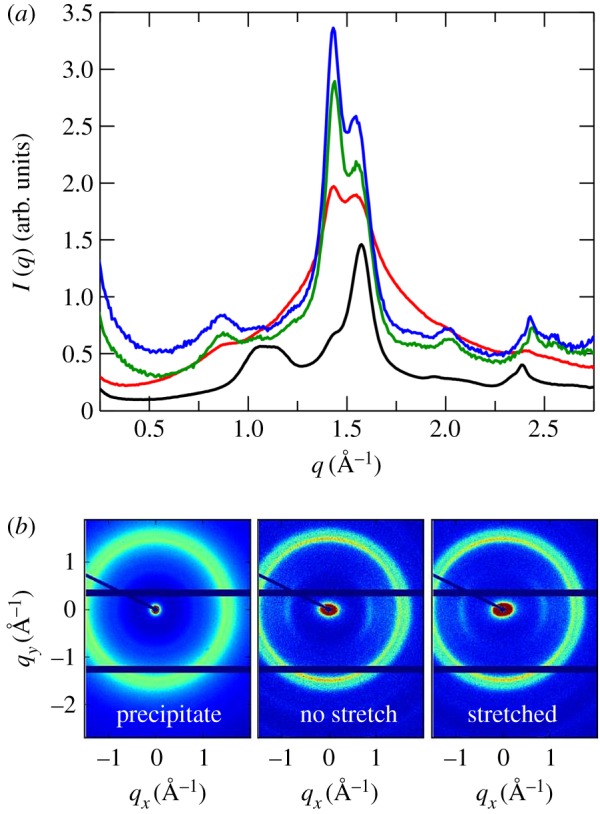
(*a*) WAXS patterns of the starting material MCC (black line), the cellulose regenerated by washing with water a stable solution of cellulose in 40 wt%TBAH(aq) (red line) and the non-stretched (green line) and stretched (blue line) fibres. (*b*) Two-dimensional WAXS patterns of direct precipitate, non-stretched fibre and stretched fibre (left to right). The fibres were aligned in the vertical direction (*y*).

The increase of water content in the solutions leads to the precipitation of cellulose, also referred to as regeneration in the fibre spinning process. WAXS was used to characterize such precipitates, and the WAXS pattern of precipitated cellulose is reported in figure 6*a*, together with that of the starting material, MCC. Peaks centred at different *q*-values are observed for the two patterns, highlighting different packings of the cellulose chains in the crystalline structures. Comparing the peak positions with the one given by the literature [26–28], it is possible to infer that the starting material has, as expected, the fingerprint of cellulose I, with the characteristic peak centred at 1.05 Å^−1^, while the process of regeneration facilitates the formation of cellulose II.

Regeneration is of importance for the production of cellulose fibres. In this process, the cellulose solution is injected through a mesh into a coagulation bath in a controlled way. Subsequent pulling on the other end leads to elongated precipitates that after drying constitute the cellulose fibres.

The experimental set-up described in the Material and methods section is a means of mimicking this process on a smaller scale with the advantage of a lower amount of required sample. To show its feasibility fibres with two different stretch factors, *f* = 1 (no stretching) and *f* = 3.5 (stretching), have been produced. In figure 6*a*,*b*, the one-dimensional and two-dimensional WAXS scattering patterns for both fibres are depicted, respectively. As expected, both patterns are more or less identical in shape and coincide with the one of cellulose II precipitate described in the previous section. It is notable that narrower signals are obtained for the fibres in comparison with the precipitated cellulose, and this is an indication of the higher crystallinity of the fibres.

In summary, we have shown that this new technique is suitable for producing fibres with a significant content of cellulose II. Therefore, this technique seems to provide an easy and quick way to check on the precipitation of cellulose into fibres, in a more controlled way than the injection with a syringe of the spin dope by hand in the coagulation bath.

### 3.6. Stable, metastable and unstable cellulose solutions

Figure 3 represents a stability map that summarizes the conclusions from our study on the dissolution state of cellulose (MCC) at various TBAH(aq) concentrations. An important basis for our understanding of this system is the relative stability of the two polymorphs cellulose I (native cellulose) and cellulose II, generally formed when cellulose is regenerated, i.e. recrystallized from solution. Cellulose I is less stable compared to cellulose II, and hence has a higher molecular solubility. This has the consequence that cellulose I, i.e. MCC, may dissolve while creating a supersaturated solution with respect to cellulose II, if the concentration is above the solubility of cellulose II. In such a supersaturated solution, cellulose aggregates as cellulose II. Estimated solubility curves for cellulose I and cellulose II are shown in figure 3.

We can define three different regimes, corresponding to stable (*S*), metastable (*M*) and unstable (*U*) cellulose solutions. In the stable regime, corresponding to cellulose concentrations below the solubility of cellulose II, cellulose is molecularly dissolved and the solutions are thermodynamically stable. For concentrations above the solubility of cellulose II, there is a metastable regime. Here, there is aggregation of cellulose, but no complete precipitation. In fact, we observe a steady state where the aggregation is finite and the solutions are kinetically stable. We ascribe this steady state to the very complex process of polymer crystallization [29,30]. Our observations are consistent with the suggestion that polymer crystallization, in contrast with the crystallization of small molecules, involves multiple free energy barriers and hence multiple metastable states [29,30]. Finally, for lower TBAH concentrations, there is an unstable regime where there is a massive aggregation and macroscopic precipitation of cellulose. Solutions of different concentrations of cellulose in 40 wt% TBAH were gradually diluted with water. At a certain point of dilution, macroscopic precipitation of cellulose was observed, marking the onset of instability. Here, the supersaturation is sufficiently large so that the system cannot be trapped in a metastable state.

### 3.7. On cellulose solubility in strong alkali

Finally, we briefly discuss cellulose solubility in strong alkali, and why TBAH(aq) appears to be a much better cellulose solvent compared to, for example, NaOH(aq). Owing to its stable crystalline state, cellulose is insoluble in water and other common solvents, from polar to non-polar, and there are only a limited number of solvents/solutions known to solubilize cellulose. Being insoluble in, for example, water means that the free energy of dissolution, Δ*G*_d_ > 0. In order for cellulose to go into solutions thus requires an additional process having a sufficiently negative free energy change. For aqueous systems, it has been known for more than a century that strong alkali is able to dissolve cellulose, and is an important component in the viscose process. Strong alkali may dissolve cellulose, because it offers a neutralization step with a negative free energy change, Δ*G*_n_ < 0. The p*K*_a_ of glucose and similar sugars is approximately 12.5–13.5 [31], and we expect similar values for cellulose although we need to take into account polyacid effects. The titration of cellobiose was recently demonstrated by electrophoretic NMR [10]. Titrating cellulose results in a negatively charged polyelectrolyte where the electrostatic potential increases slightly the p*K*_a_. Hence, cellulose –OH groups display a range of p*K*_a_ values, not only because of the different sites along the polymer but also because of the polyacid effect. Nevertheless, if the pH is sufficiently high, higher than typical p*K*_a_ values, there is sufficient neutralization and corresponding negative Δ*G*_n_ to compensate for Δ*G*_d_ > 0, so that for the combined process, Δ*G *= Δ*G*_d_ + Δ*G*_n_ < 0. This view implies that cellulose solubility increases with increasing pH, which is consistent with our findings that cellulose is more soluble in 55 wt% TBAH(aq) compared to 40 wt% TBAH(aq).

Concentrated NaOH(aq) is a classical cellulose solvent. However, cellulose can only be solubilized within a narrow concentration interval of NaOH. Typically, cellulose can be solubilized in 2 M NaOH(aq) but not in 1 M and not in 3 M. This narrow solubility window can be understood as follows. At lower NaOH concentrations, Δ*G*_n_ is not sufficiently negative and the solution is unstable with respect to cellulose I that cannot dissolve. At higher NaOH concentrations, on the other hand, cellulose precipitates in the form of polyelectrolyte sodium salts [5]. Here, we have to consider the solubility product *K*_s_= [cellulose][Na^+^]^*n*^, where *n* is the effective valency of the cellulose ion. As *n* is typically a high number, this implies that the solubility of the sodium salt decreases strongly with increasing NaOH concentration. As a consequence, solubilizing cellulose is only possible within a narrow window of NaOH concentrations.

Replacing the small Na^+^ ions with the bulky TBA^+^ has the important consequence of destabilizing the cellulose salt crystal. TBA^+^ is too bulky to fit properly into a crystalline lattice, which is also why many TBA^+^ salts, in fact, are ionic liquids.

## 4. Conclusion

MCC solutions in TBAH(aq) at various concentrations of cellulose and TBAH were studied by means of turbidity and SAXS techniques. As a first result, 55 wt% TBAH(aq) was found to solubilize cellulose in larger amounts than the more diluted solvent, 40 wt%. Moreover, a stability map of the cellulose/TBAH/water system was developed. Stable solutions of cellulose were obtained for cellulose concentrations below the solubility of cellulose II, the most stable crystalline polymorph. Microscopic precipitate, i.e. aggregates, which indicates the metastability of the system, was detected as soon as the solubility curve of cellulose II was crossed. Instability, i.e. the massive crystalliztion of cellulose, led to the presence of macroscopic precipitate, and occurred with a further decrease in the cellulose concentration. Such regenerated cellulose was characterized by WAXS, and the analysis was led further using a custom-built step-motor-driven syringe pump system to mimic the wet spinning process on a small scale, as a first example of a laboratory-scale method for scanning the properties of spinning dopes in fibre formation processes and an easy way to check the properties of regenerated fibres.

The question of the reason for the solubility of cellulose in TBAH(aq) was also addressed, and the combination of high pH, which favours the deprotonation of the cellulose chains, and the bulkiness of the TBA^+^ cation, which hinders precipitation and crystallization, seems to be the key point for the effectiveness of TBAH(aq) in solubilizing cellulose.

## Supplementary Material

Cellulose Stability_Supplementary material.pdf

## Data accessibility

Turbidity, SAXS and WAXS data are deposited at Dryad (http://dx.doi.org/10.5061/dryad.gv3vg) [32]. Light microscope images of solutions of cellulose II in 40 wt% TBAH(aq) are provided as electronic supplementary material.
