# Supplementary material for: Stable, metastable and unstable cellulose solutions
Source: R Soc Open Sci. 2017 Aug 30;4(8):170487. doi: 10.1098/rsos.170487 (PMC5579112; doi:10.1098/rsos.170487)
Supplement: Cellulose Stability_Supplementary material.pdf [file rsos170487supp1.pdf]

Supplementary material for

Stable, metastable and unstable cellulose solutions

Marta Gubitosi, Pegah Nosrati, Mona Koder Hamid, Stefan Kuczera, Manja A. Behrens,*

Eric G. Johansson, Ulf Olsson

Physical Chemistry, Lund University, Box 124, SE-221 00 Lund, Sweden

marta.gubitosi@fkem1.lu.se

Light microscopy. Solubility of MCC and regenerated cellulose in 40 wt% TBAH(aq) was investigated by light microscopy. Solutions were prepared accordingly to the previous sections descriptions in the concentration ranges 0.001-0.030 g/cm^3. Solubility tests were performed using a Zeiss Axioplan microscope. A drop of cellulose solution (ca. 50 µl) was placed on a microscope slide and covered with a 24 x 40 mm cover glass. The images were recorded by a Zeiss Axiocam MRc camera connected to the microscope. Magnifications of 10 x and 100 x were used.

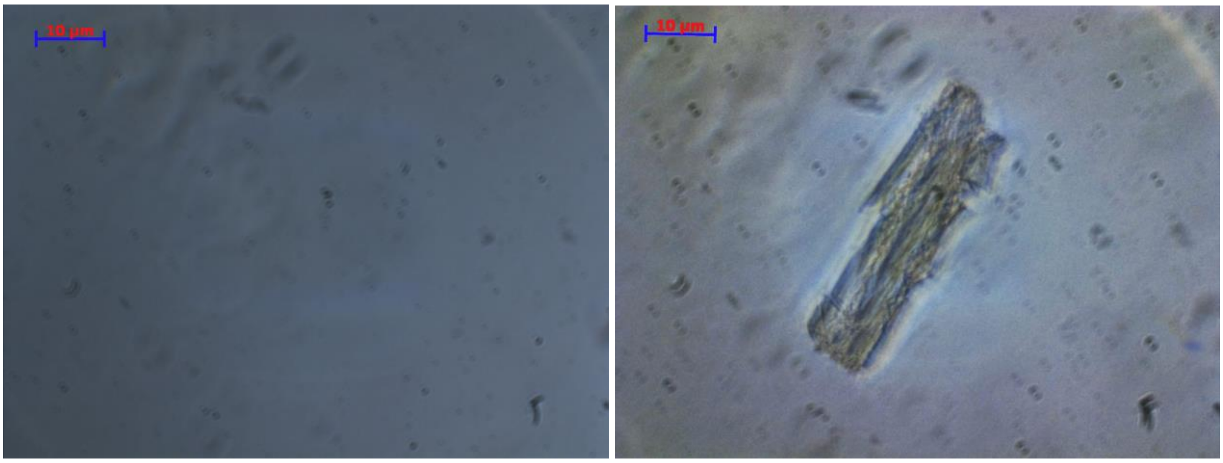

Figure S1. Light microscopy image of 0.002 (left panel) and 0.0025 g/cm^3 (right panel) regenerated cellulose (cellulose II) in 40 wt.% TBAH(aq) with a magnification of 100x.
